# Optimization and application of genome prediction model in rapeseed: flowering time, yield components, and oil content as examples

**DOI:** 10.1093/hr/uhaf115

**Published:** 2025-04-30

**Authors:** Wenkai Yu, Xinao Wang, Hui Wang, Wenxiang Wang, Hongtao Cheng, Desheng Mei, Lixi Jiang, Qiong Hu, Jia Liu

**Affiliations:** Key Laboratory for Biology and Genetic Improvement of Oil Crops, Oil Crops Research Institute of Chinese Academy of Agricultural Sciences, Ministry of Agriculture and Rural Affairs, Wuhan 430062, China; Key Laboratory for Biology and Genetic Improvement of Oil Crops, Oil Crops Research Institute of Chinese Academy of Agricultural Sciences, Ministry of Agriculture and Rural Affairs, Wuhan 430062, China; Shenzhen Graduate School, Chinese Academy of Agricultural Sciences, No. 7 Pengfei Road, Longgang District, Shenzhen 518100, China; Key Laboratory for Biology and Genetic Improvement of Oil Crops, Oil Crops Research Institute of Chinese Academy of Agricultural Sciences, Ministry of Agriculture and Rural Affairs, Wuhan 430062, China; Key Laboratory for Biology and Genetic Improvement of Oil Crops, Oil Crops Research Institute of Chinese Academy of Agricultural Sciences, Ministry of Agriculture and Rural Affairs, Wuhan 430062, China; Key Laboratory for Biology and Genetic Improvement of Oil Crops, Oil Crops Research Institute of Chinese Academy of Agricultural Sciences, Ministry of Agriculture and Rural Affairs, Wuhan 430062, China; Key Laboratory for Biology and Genetic Improvement of Oil Crops, Oil Crops Research Institute of Chinese Academy of Agricultural Sciences, Ministry of Agriculture and Rural Affairs, Wuhan 430062, China; Institute of Crop Science, Zhejiang University, Hangzhou 310058, China; Key Laboratory for Biology and Genetic Improvement of Oil Crops, Oil Crops Research Institute of Chinese Academy of Agricultural Sciences, Ministry of Agriculture and Rural Affairs, Wuhan 430062, China; Key Laboratory for Biology and Genetic Improvement of Oil Crops, Oil Crops Research Institute of Chinese Academy of Agricultural Sciences, Ministry of Agriculture and Rural Affairs, Wuhan 430062, China

## Abstract

Rapeseed is the second largest oilseed crop in the world with short domestication and breeding history. This study developed a batch of genomic prediction models for flowering time (FT), oil content, and yield components in rapeseed. Using worldwide 404 breeding lines, the optimal prediction model for FT and five quality and yield traits was established by comparison with efficient traditional models and machine learning (ML) models. The results indicate that quantitative trait loci (QTLs) and significant variations identified by genome-wide association study (GWAS) can significantly improve the prediction accuracy of complex traits, achieving over 90% accuracy in predicting FT and thousand grain weight. The Genomic Best Linear Unbiased Prediction (GBLUP) and Bayes–Lasso models provided the most accurate prediction overall, while ML models such as GBDT (Gradient-Boosting Decision Trees) exhibited strong predictive performance. Our study provides genome selection solution for the high prediction accuracy and selection of complex traits in rapeseed breeding. The use of a diverse panel of 404 worldwide lines ensures that the findings are broadly applicable across different rapeseed breeding programs.

## Introduction

Rapeseed (*Brassica napus L*) is the second largest oilseed crop in the world and serves as a major source of edible vegetable oil [1]. As an allotetraploid, rapeseed has two subgenomes derived from *Brassica rapa* and *Brassica oleracea* [2]. It shares a close evolutionary relationship with the model plant *Arabidopsis* of the *Brassicaceae* family. The rapeseed genome is characterized by extensive rearrangements and recombinations [3], which makes it an excellent model for studying polyploidy in plants [4]. With the decreasing costs of genome sequencing and the availability of high-quality reference genomes, researchers are now able to perform comprehensive genomic and genetic analyses of traits such as yield [5], seed quality [6], plant architecture [7], and stress tolerance [8]. To date, over 130 functional genes have been cloned in rapeseed [9]. With the development of new technologies, rapeseed research has gradually integrated advanced methods such as marker-assisted breeding, transgenic breeding, gene editing, and genomic selection [10], significantly improving breeding efficiency.

During domestication, rapeseed has evolved into three ecotypes: spring, winter, and semiwinter types with different requirement of low-temperature vernalization. *Brassica napus* originated in Europe as a winter type that requires an extended vernalization period to flower [11]. It was subsequently introduced to other regions, including China, Australia, Canada, and India, where it further differentiated into spring and semiwinter types to adapt to diverse environmental and climatic conditions [12, 13]. Flowering time (FT) is a major factor in rapeseed adaptation to climate and is also a key determinant of yield [14]. The number of siliques in rapeseed is significantly influenced by germination timing and bud number [15, 16], which in turn determines seed yield potential [17]. Flowering is affected by vegetative growth duration and low temperatures during vernalization [18–20]. FT represents the transition from vegetative to reproductive growth and is closely related to plant height, silique number (SN), and seed yield [21]. In *Arabidopsis*, 180 genes have been identified as regulators of FT [22]. In rapeseed, chromosomal duplication and recombination have resulted in even more regulatory genes, which makes flowering regulation a complex network [23]. Several key genes and their homologs determine FT in rapeseed, including *CO* [24], *FT* [25], *VIN3* [26], and *FLOWERING LOCUS C (FLC)* [27]. Selection for appropriate FT is a crucial step in rapeseed breeding. However, due to the complexity of the regulatory network involving multiple genes, it is insufficient to select based on only one or two genes. Genomic selection provides a feasible approach to breeding rapeseed lines with optimal FT. By combining FT and yield traits using genomic selection, it is possible to rapidly aggregate beneficial alleles from different loci and improve rapeseed lines.

Genomic prediction is a prebreeding method based on markers covering the entire genome [28]. Increasing marker density across the genome generally improves prediction accuracy. Genomic prediction incorporating high-density markers and environmental data has been widely applied in wheat, maize, and legumes [29–32]. Compared to single-environment prediction models, adding multienvironment data has improved prediction accuracy by 10%–40% in these crops. Genome resequencing data provide almost saturated genotypic information on genomic variation, but many of these variations are redundant for genomic prediction. Only genotypic variations associated with phenotypic effects can significantly enhance prediction performance [33, 34]. Over the past decade, genomic prediction models have been used for breeding in crops such as maize, wheat, barley, and oats [35–38]. By integrating rapid breeding techniques with genomic prediction, it is possible to achieve six generations of wheat per year and facilitate the selection of traits such as plant height, FT, and yield components at the seedling stage [39]. Genomic prediction based on superior genotypes has proven reliable across different environments [39–43].

Previous genomic prediction studies in rapeseed have focused on populations like DH, F2, and NAM. Würschum *et al*. [44] used 391 DH lines to predict traits such as oil content (OC), protein content, plant height, and FT using RR-BLUP, achieving prediction accuracy of 50% for oil and protein contents and 80% for plant height and FT. Zou *et al*. [45] developed a genomic selection model using a permanent F_2_ population, achieving prediction accuracy of 76% for OC and 66% for protein content using Best Linear Unbiased Prediction (BLUP). Roy *et al*. [46] used 337 rapeseed lines to develop models to predict stem rot resistance, with prediction accuracy ranging from 42% to 67%. Knoch *et al*. [47] used GBLUP (Genomic Best Linear Unbiased Prediction) and RKHS models to predict yield, seed quality, and plant architecture, with prediction accuracy ranging from 27% to 70%. Perumal *et al*. [48] used a NAM population to predict complex traits such as FT, maturity, plant height, and thousand-seed weight (TSW), achieving accuracy of 27%–71%. Previous studies have extensively explored genomic selection for rapeseed traits related to quality, yield, and disease resistance. However, there is still a need for improvement on selection of genetic population, molecular markers, and prediction models to further enhance prediction accuracy.

Base on the purpose, this study will explore the prediction efficiency and accuracy of traditional models and machine learning (ML) models for the complex traits. Additionally, it will deliberate on the impact of genome-wide association study (GWAS) loci with different threshold on the accuracy of the genomic prediction (GP) models. This study collected genotypic and phenotypic data from 404 rapeseed breeding lines from the major cultivation regions worldwide. The research goal was to analyze the genetic architecture of complex traits such as FT, yield, and quality in rapeseed, which included OC, TSW, silique length (SL), number of seeds per silique (NSS), and SN. Seven genome prediction models, including traditional and ML models, were compared to better GP accuracy. Genotypic data were processed using principal component analysis (PCA), CDS variants, and GWAS threshold-associated variants to determine the optimal prediction model and input features. The results provide a highly accurate genomic prediction framework for complex traits like FT, quality, and yield components in rapeseed and will give the route to integrate the GP pipeline to rapeseed breeding program.

## Results

### Genotypic data analysis

**Figure 1:**
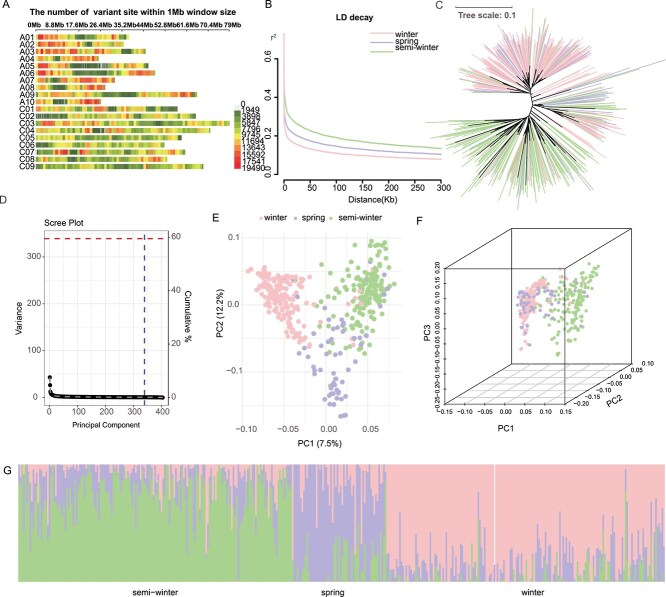
Distribution of genetic variants and population structure in rapeseed. Filtered variants were used to generate the following plots: chromosome distribution of genetic variants, genome-wide LD decay, population structure analysis, and PCA. Winter-type accessions are indicated in pink, semiwinter-type accessions in green and spring-type accessions in purple. (A) Chromosomal distribution of genetic variants: The number of variants per 1 MB was used for statistical analysis, with red indicating regions of high variant density and green indicating regions of low variant density. (B) Genome-wide LD decay curve: The curve illustrates the rate of linkage disequilibrium decay across the genome. (C) Phylogenetic tree: a phylogenetic tree based on genotypic data of 404 rapeseed accessions. (D) Principal component variance explained: variance explained by the first three principal components in the analysis. (E) 2D PCA Plot: A 2D PCA plot based on genotypic data, using the first three principal components for analysis. (F) 3D PCA plot: A 3D PCA plot showing the genotypic variance of the accessions. (G) Population structure plot: The population structure of the 404 accessions as determined by genotypic clustering.

The genomic variation across 404 rapeseed accessions was comprehensively analyzed. After mapping resequencing data to the reference genome, variants were filtered to exclude single nucleotide polymorphisms (SNPs) and InDels with >10% missing data and those with minor allele frequencies <0.05, resulting in a total of 23 459 926 variants. This included 19 077 712 SNPs and 4 382 224 InDels, with their distribution across 19 chromosomes (Fig. 1A). The A subgenome contained 10 047 520 variants, while the C subgenome had 13 412 416 variants. Linkage disequilibrium (LD) decay was calculated using these variants (Fig. 1B). Setting the threshold at *R^2^* = 0.2 [49], the LD decay distance for the entire genome was ~52 039 bp, which was used to define effective GWAS regions. Among the three ecotypes, the winter type showed the shortest LD decay distance, followed by the spring type, and the semiwinter type had the longest distance. This LD decay pattern reflects the domestication history of these ecotypes. As the spring and semiwinter types emerged later, they contain fewer recombination events, leading to longer LD decay distances, which is consistent with the domestication history of *B. napus* (Fig. 1B). The missing genotype data were imputed using the BEAGLE software. Previous studies have demonstrated that such imputation can improve genomic prediction accuracy [50].

### Population structure analysis

Among the 404 rapeseed lines, 199 were from Europe, with the highest representation from Germany (84 lines). Poland and Sweden contributed 23 lines each, while 64 lines were from France, the UK, Russia, and the Netherlands. The remaining 176 lines originated from Asia, with 151 from China (accounting for 85.6%), and the others from Pakistan, South Korea, Japan, and other countries. There were also 17 lines from Oceania, mostly from Australia (16 lines), and 7 lines from the Americas, primarily from Canada. Additionally, there was a single line from Morocco in Africa. There were 165 semiwinter lines (40%), 61 spring lines (16%), and 178 winter lines (44%). Winter types were predominantly from Europe, semiwinter types from Asia, and spring types mainly from Australia (Supplementary Table S1). Phylogenetic analysis (Fig. 1C) showed winter types clustered at the top of the phylogenetic tree, semiwinter types at the bottom, and spring types in the middle. PCA of the genotypic data revealed that the first three principal components explained a significant proportion of the genetic variance (Fig. 1D), requiring 321 components to reach 95% cumulative variance. The 2D PCA plot (Fig. 1E) showed distinct clustering of winter, semiwinter, and spring types at three corners of a triangle. The 3D PCA plot (Fig. 1F) showed clear separation of winter and semiwinter types on either side, with spring types positioned in the middle. The population structure plot (Fig. 1G) further illustrated clear differences between the three ecotype subgroups (Supplementary Table S2). The constructed phylogenetic tree and population structure analysis demonstrated significant genetic divergence among the different rapeseed ecotypes, which could be distinctly separated using genotypic data matrices, allowing accurate simulation of population relationships.

### Phenotypic analysis

**Figure 2:**
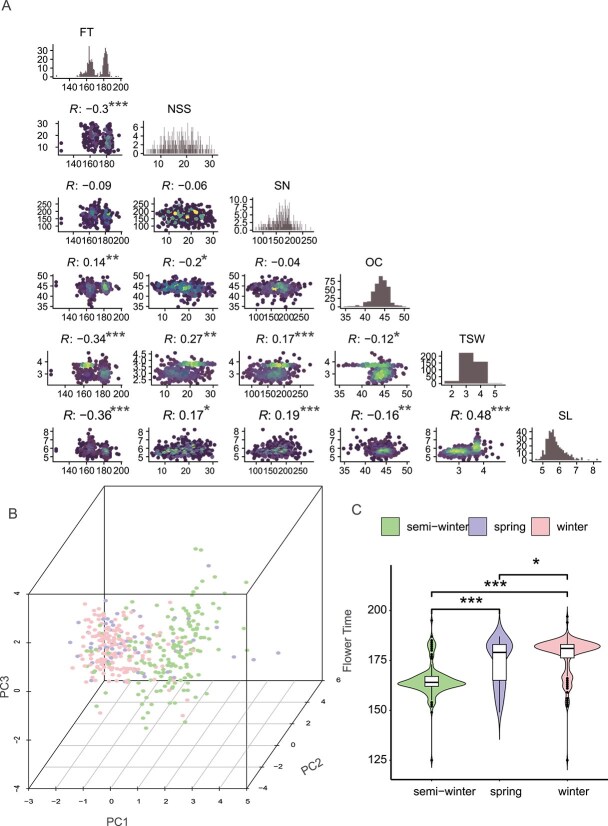
Phenotypic distribution of rapeseed. (A) Distribution and correlation plot of traits: The distribution of six traits in the rapeseed population, with the Pearson correlation coefficient (*R*) among the traits. (B) PCA of six traits: PCA based on the first three principal components of the six phenotypic traits. Different types of rapeseed accessions are color-coded: green for semiwinter-type, purple for spring-type, and pink for winter-type accessions. (C) Violin plot of flowering time distribution. Distribution of flowering time across the three types of rapeseed*.* It indicates significant correlations using a two-tailed *t*-test (^*^P < .05; ^*^^*^P < .01; ^*^^*^^*^P < .001).

Phenotypic data for agronomic traits were collected over 2 years and analyzed to calculate the BLUPs, which were used as stable phenotypes for GWAS and genomic prediction. Among the rapeseed accessions, OC, SN, NSS, SL, and TSW exhibited unimodal normal distributions, while FT showed a bimodal distribution (Fig. 2A). The two peaks of FT were at 160 and 180 days, averaging 172 days, a maximum of 197 days, and a minimum of 125 days, with a standard deviation (SD) of 10.89 (Table 1). The violin plot (Fig. 2C) showed that the FT of semiwinter lines averaged 163 days, whereas that of spring and winter types averaged 180 days. The difference in FTs between semiwinter lines and spring or winter lines was highly significant (Fig. 2C). OC ranged from 34.79% to 49.79%, with a mean of 43.86% and SD of 2.08 (Table 1). SL varied from 4.58 to 8.22 cm, with an average of 5.73 cm and SD of 0.53. SL was weakly negatively correlated with FT (correlation coefficient = −0.36) and moderately positively correlated with TSW (correlation coefficient = 0.48). SN ranged from 72 to 280, with a mean of 169.70 and SD of 36.00. NSS ranged from 5 to 30, with a mean of 17.60 and SD of 5.72. NSS was negatively correlated with FT and OC (correlation coefficients of −0.30 and − 0.20, respectively) and positively correlated with TSW (correlation coefficient = 0.27). TSW ranged from 2.20 to 4.74 g, with a mean of 3.26 g and SD of 0.47. TSW was negatively correlated with FT (correlation coefficient = −0.34). FT showed weak negative correlations with NSS, TWS, and SL and no significant correlations with SN and OC. Based on PCA of the phenotypic data (Fig. 2C), there were differences between semiwinter and winter types, while most spring types showed phenotypic characteristics closer to those of winter types.

**Table 1:** Statistical summary of BLUPs data for six traits in the panel of 404 rapeseed accessions

Trait name	Max	Min	Mean	Variance	SD
FT	197	125	171.86	118.56	10.89
OC	49.79	34.79	43.86	4.31	2.08
SL	8.22	4.58	5.73	0.28	0.53
SN	280.50	72.00	169.70	1295.83	36.00
NSS	30.67	4.57	17.60	32.64	5.72
TSW	4.74	2.21	3.26	0.22	0.47

### GWAS analysis

**Figure 3:**
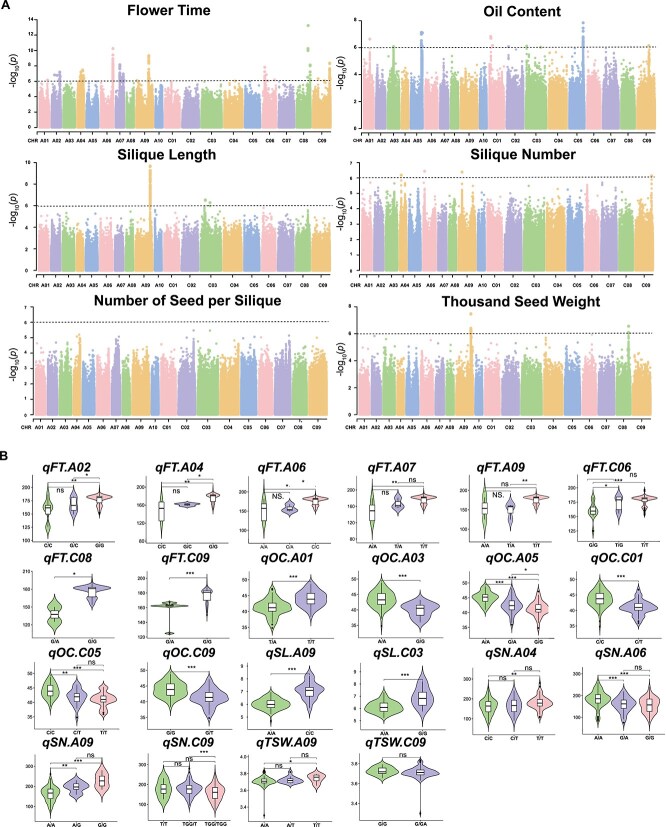
Genome-wide association study of six traits. (A) Manhattan Plots for FT, OC, SL, SN, NSS, and TSW. Manhattan plots showing the association of genetic loci with the six traits in the rapeseed population. The *Y*-axis represents the effect value of variants, with –log10(P) as the vertical axis, and the *X*-axis corresponds to the 19 chromosomes of rapeseed. The dashed line represents the significance threshold (−log10(*P*) = 6). (B) Haplotype plots of peak variants for each QTL. Statistical significance of haplotype effects is determined by a *t*-test, with ^*^P < .05; ^*^^*^P < .01; and ^*^^*^^*^P < .001.

**Figure 4:**
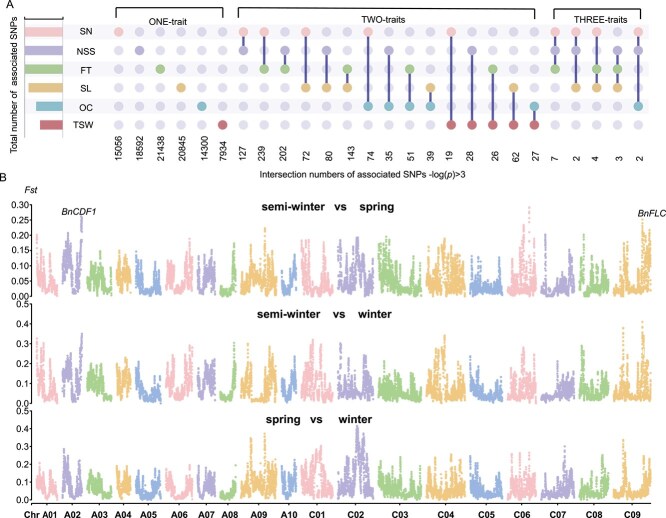
Overlapping GWAS results and population differentiation. (A) Upset Venn diagram showing the overlap number of the SNPs associated with the six traits. Venn diagram illustrating the loci with –log10(*P*) > 3 that overlap across the six traits studied in the rapeseed population. The number of overlapping SNPs for each pairwise comparison is shown below the diagram. The *X*-axis represents the overlapping SNPs associated with these traits, while the *Y*-axis presents the six traits with total associated SNPs. (B) Differentiation Coefficients (*Fst*) across chromosomes for the three subgroups (spring, semiwinter, and winter): The *Fst* values across the 19 chromosomes, representing population differentiation among the three rapeseed subgroups. The *X*-axis corresponds to chromosome numbers, and the *Y*-axis shows the *Fst* values, highlighting regions of high differentiation between subgroups.

Using BLUPs calculated for agronomic traits, we performed a GWAS. The significance threshold for association analysis was set at –log10(*p*) ≥ 6 using Bonferroni correction (Supplementary Fig. S1). A total of 22 significant quantitative trait locus (QTL) regions were identified with 13 QTLs on A genome and 9 QTLs on C genome (Table 2, Fig. 3A). Eight significant QTLs for FT were identified on chromosomes A02, A04, A06, A07, A09, C06, C08, and C09. Among these, *qFT.C08* had the highest effect value of 5.79E-14, while stable peaks with –log10(*p*) > 8 were observed in *qFT.A06*, *qFT.A09*, and *qFT.C09*. Six significant QTLs for OC were identified on chromosomes A01, A03, A05, C01, C05, and C09, with *qOC.C08* having the highest effect value of 1.44E-08. Two significant QTLs for SL were on A09 and C03, with *qSL.A09* having an effect value of 1.96E-10. Four significant QTLs for SN were on A04, A06, A09, and C09. TSW had two significant QTLs on A09 and C08, with effect values of 3.27E-08 and 2.78E-07, respectively. Of the 22 QTLs, all but *qTSW.C09* showed significant haplotype differences (Fig. 3B) (Supplementary Table S3). An analysis of loci with –log10(*p*) > 3 identified 96 959 loci, with FT sharing the most overlapping loci with other traits, including 239 loci overlapping with SN, 202 with NSS, and 143 with SL (Fig. 4A). FT influences bud development and silique differentiation, ultimately affecting yield. This result aligns with our findings, as FT had the most overlapping loci with silique-related traits. SL shared the most overlapping loci with TSW (62 loci), which is consistent with the high correlation between these two traits (Fig. 2B). Additionally, *Fst* analysis revealed stable differentiation regions (*Fst* > 0.25) on A02, A09, C06, and C09 (Fig. 4B), indicating high levels of domestication in these regions. The high differentiation region on A02 overlapped with *qFT.A02* identified for FT, A09 overlapped with *qSL.A09* for SL, and C09 overlapped with *qFT.C09* and *qSN.C09* for FT and SN, respectively (Fig. 4B).

**Table 2:** The information of the QTL associated with FT, OC, SL, SN, and TSW by GWAS in this study

Trait	QTL	Chr	Peak position^a^	*P*-value	QTL range^b^	Candidate genes ID
FT	*qFT.A02*	A02	31103911	6.31E−08	31051911–31155911	*BnaA02G0352400ZS*
	*qFT.A04*	A04	22846166	6.33E−09	22794166–22898166	*BnaA04G0248600ZS*
	*qFT.A06*	A06	47559072	5.57E−11	47507072–47611072	*BnaA06G0433300ZS*
	*qFT.A07*	A07	16408859	1.25E−11	16356859–16460859	*BnaA07G0122900ZS*
	*qFT.A09*	A09	48835744	4.62E−10	48783744–48887744	*BnaA09G0427800ZS*
	*qFT.C06*	C06	7993765	1.51E−08	7941765–8045765	*BnaC06G0021600ZS*
	*qFT.C08*	C08	44432255	5.79E−14	44380255–44484255	
	*qFT.C09*	C09	64831981	4.33E−09	64779981–64883981	*BnaC09G0556100ZS* *BnaC09G0556700ZS* *BnaC09G0557000ZS*
OC	*qOC.A01*	A01	23099126	2.33E−07	23047126–23151126	
	*qOC.A03*	A03	22570735	8.40E−07	22518735–22622735	*BnaA03G0416300ZS*
	*qOC.A05*	A05	41113713	7.35E−08	41061713–41165713	
	*qOC.C01*	C01	6005735	1.50E−07	5953735–6057735	*BnaC01G0095100ZS*
	*qOC.C05*	C05	54016444	1.44E−08	53964444–54068444	*BnaC05G0501300ZS*
	*qOC.C09*	C09	44501778	7.04E−07	44449778–44553778	
SL	*qSL.A09*	A09	57148997	1.96E−10	57096997–57200997	*BnaA09G0560100ZS*
	*qSL.C03*	C03	17931133	2.76E−07	17879133–17983133	*BnaC03G0281100ZS*
SN	*qSN.A04*	A04	5536474	6.59E−07	5484474–5588474	*BnaA04G0068600ZS*
	*qSN.A06*	A06	5679650	3.70E−07	5627650–5731650	*BnaA06G0094500ZS*
	*qSN.A09*	A09	13303680	4.15E−07	13251680–13355680	*BnaA09G0194900ZS*
	*qSN.C09*	C09	63093434	7.34E−07	63041434–63145434	
TSW	*qTSW.A09*	A09	57502874	3.27E−08	57450874–57554874	*BnaA09G0562700ZS*
	*qTSW.C08*	C08	46461951	2.78E−07	46409951–46513951	*BnaC08G0412500ZS*

a The physical positions were referenced to the ZS11 reference genome.

b The criterion for QTL identification was set at –log10(p) > 6. With an *r*^2^ threshold of 0.2, the genome-wide LD decay distance was 52 kb, and the QTL regions were expanded by 52 kb upstream and downstream of the peak SNP positions.

### Genomic prediction model construction

**Figure 5:**
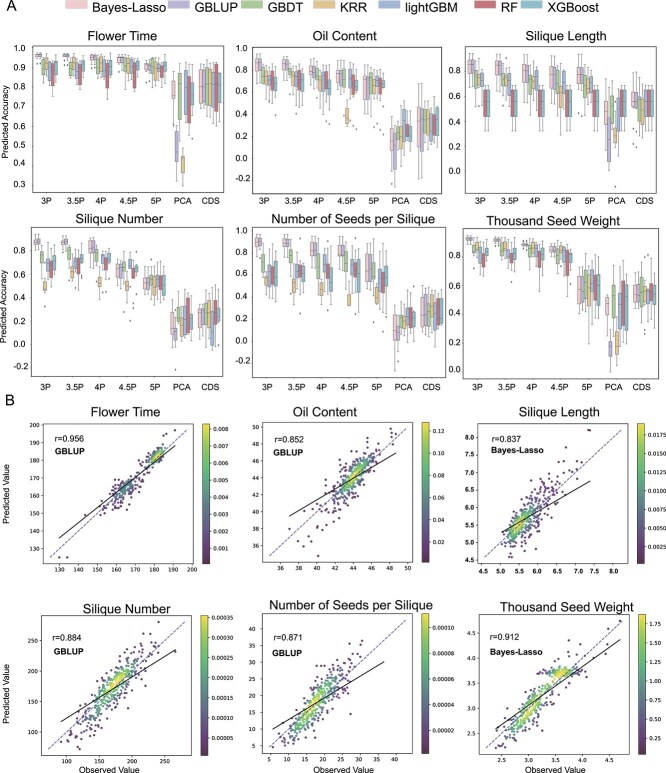
Genomic prediction accuracy for six traits using seven input features. (A) Boxplot of prediction accuracies from 10-fold cross-validation. The *Y*-axis represents prediction accuracy, expressed as the Pearson correlation coefficient between predicted and actual values for each fold. The *X*-axis represents the seven input features, using Bayes–Lasso, GBLUP, GBDT, KRR, LightGBM, RF, and XGBoost models. (B) Correlation plot of all prediction results from the optimal model for each trait using 10-fold cross-validation. The *Y*-axis represents the predicted values, and the *X*-axis represents the actual values. The color of each point indicates density in that area, with yellow indicating higher density.

We performed PCA for dimensionality reduction, alongside random selection of variants in CDS regions and high-effect variants from GWAS for input data. Ten-fold cross-validation was used to assess prediction accuracy. For FT, the GBLUP model achieved the highest prediction accuracy (Fig. 5A). With effect values of 3 and 3.5, prediction accuracy reached >95.5% with no substantial increase. At effect value of 4 and 4.5, prediction accuracy of 94.4% and 93.5% was achieved, respectively. For CDS-derived features and PCA-reduced features, ML models performed more effectively, with Light Gradient Boosting Machine (LightGBM) and Gradient-Boosting Decision Trees (GBDT) achieving 77.8% and 80.7% accuracy, respectively (Table 3). FT is relatively easy to predict, and an effect value of 4 was sufficient for accurate prediction. Scatter plots of predicted values versus actual values for each fold of cross-validation using the optimal model (effect value 3) showed that predicted FT values were concentrated ~160 and 180 days, which is consistent with FT distribution of the population. Most points were located near the diagonal, effectively distinguishing accessions with different FTs for selecting three types of rapeseed lines (Fig. 5B).

**Table 3:** Prediction accuracy for genomic prediction models of traits using seven models of Bayes–Lasso, GBLUP, GBDT, KRR, LightGBM, RF, and XGBoost under feature numbers of different GWAS effect values (3P, 3.5P, 4P, 4.5P, and 5P), PCA and CDS-derived

Feature	Model	FT	OC	SL	SN	NSS	TSW
3P	Feature number	21 438	14 300	20 845	15 056	18 592	7934
	BL	95.70%^a^	84.06%	83.77%	86.80%	89.20%	92.38%
	GBLUP	95.78%	84.39%	83.90%	87.53%	89.24%	92.32%
	GBDT	89.40%	75.45%	73.77%	74.38%	71.62%	84.21%
	KRR	91.83%	72.49%	73.47%	49.49%	54.69%	86.74%
	lightGBM	88.04%	69.36%	72.16%	68.88%	60.82%	81.27%
	RF	86.27%	69.90%	53.73%	65.39%	61.48%	78.30%
	XGBOOST	90.09%	66.39%	53.73%	70.79%	60.26%	83.55%
3.5P	Feature number	12 595	8465	12 420	7761	10 837	4466
	BL	95.42%	83.86%	82.32%	86.47%	88.70%	91.42%
	GBLUP	95.42%	83.67%	82.08%	87.13%	88.49%	91.41%
	GBDT	89.30%	77.18%	72.44%	75.92%	74.38%	85.16%
	KRR	91.99%	72.68%	73.72%	62.68%	52.15%	86.81%
	lightGBM	88.02%	68.81%	70.23%	68.67%	64.19%	81.61%
	RF	85.94%	69.17%	53.73%	66.49%	61.92%	77.86%
	XGBOOST	90.63%	67.61%	53.73%	72.19%	60.09%	82.33%
4P	Feature number	4280	3105	4733	1969	3420	1004
	BL	94.38%	79.51%	77.43%	81.89%	82.90%	88.58%
	GBLUP	94.48%	78.26%	77.20%	82.13%	82.78%	88.31%
	GBDT	90.22%	74.89%	69.04%	75.09%	67.77%	83.58%
	KRR	91.46%	67.18%	71.69%	52.82%	46.31%	86.23%
	lightGBM	89.25%	69.24%	71.42%	71.14%	69.20%	83.61%
	RF	84.72%	67.11%	53.73%	67.13%	62.34%	77.01%
	XGBOOST	90.18%	61.90%	53.73%	72.83%	55.37%	81.04%
4.5P	Feature number	2712	2137	3313	439	2146	557
	BL	93.59%	75.68%	75.23%	62.77%	80.27%	85.04%
	GBLUP	93.69%	73.61%	74.72%	62.64%	80.03%	84.86%
	GBDT	90.27%	70.52%	69.86%	63.38%	70.88%	80.56%
	KRR	90.61%	58.37%	59.61%	49.22%	38.52%	83.93%
	lightGBM	90.20%	69.49%	69.87%	63.34%	66.43%	81.14%
	RF	86.24%	65.29%	53.73%	57.13%	63.39%	75.01%
	XGBOOST	90.40%	63.66%	53.73%	63.81%	60.15%	78.34%
5P	Feature number	1141	759	1451	100	913	74
	BL	90.13%	65.37%	75.48%	52.93%	66.44%	56.71%
	GBLUP	89.87%	65.51%	75.18%	53.09%	66.45%	56.74%
	GBDT	89.89%	67.78%	67.11%	55.42%	59.55%	61.89%
	KRR	88.40%	63.39%	68.29%	49.64%	41.38%	55.49%
	lightGBM	89.73%	66.45%	69.52%	54.84%	56.19%	60.85%
	RF	86.70%	63.99%	53.73%	52.36%	53.55%	59.33%
	XGBOOST	90.35%	62.52%	53.73%	50.88%	55.33%	57.81%
PCA	Feature number	321	321	321	321	321	321
	BL	75.94%	13.45%	46.65%	17.69%	9.88%	44.37%
	GBLUP	47.78%	6.66%	29.11%	9.03%	6.13%	15.95%
	GBDT	75.96%	22.13%	51.47%	26.77%	19.51%	49.67%
	KRR	41.24%	18.51%	28.52%	15.95%	18.25%	19.98%
	lightGBM	77.82%	29.97%	45.10%	22.47%	22.74%	44.50%
	RF	75.24%	25.55%	53.73%	25.90%	22.14%	49.69%
	XGBOOST	75.80%	22.94%	53.73%	20.63%	20.96%	45.96%
CDS	Feature number	20 345	20 345	20 345	20 345	20 345	20 345
	BL	80.37%	27.76%	54.73%	22.51%	26.64%	54.47%
	GBLUP	80.46%	27.47%	53.95%	24.40%	27.29%	54.59%
	GBDT	80.72%	34.36%	52.70%	25.35%	25.98%	54.38%
	KRR	79.86%	32.98%	48.46%	25.84%	30.51%	53.03%
	lightGBM	78.51%	27.63%	53.73%	15.46%	22.51%	53.63%
	RF	79.26%	31.93%	53.73%	26.67%	25.58%	55.38%
	XGBOOST	78.55%	32.25%	53.73%	25.09%	26.84%	52.96%

a The prediction accuracy represents the average accuracy across all folds after performing 10-fold cross-validation.

For OC, prediction accuracies were 84.3% and 83.8% with effect values of 3 and 3.5, respectively, with little difference. At –log10(*p*) ≥ 4, prediction accuracy dropped <80%. Additionally, neither PCA data nor CDS-derived variants could predict OC with a maximum accuracy of 32.2%. For SL, prediction accuracies were 83.8% and 82.3% at effect values of 3 and 3.5, with accuracy <80% for other thresholds. For SN, NSS and TSW, the highest prediction accuracies were 87.5%, 89.2%, and 92.4%, respectively. Among these traits, prediction accuracy was similar at effect values of 3 and 3.5.

Thus, for OC, SL, SN, NSS, and TSW, prediction accuracy was similar at effect values of 3 and 3.5, but the number of variants required different significantly. For example, the variants for OC could be reduced from 14 300 to 8465, and those for SN could be reduced from 15 056 to 7761. Using an effect value of 3.5 provides better economic efficiency by requiring fewer loci for similar accuracy. For yield and quality-related traits, prediction accuracy exceeded 83% (Table 3, Fig. 5B), with most points fitting well along the diagonal, demonstrating good predictive performance.

## Discussion

Rapeseed (*B. napus*) originated in Europe and the large-scale breeding program of rapeseed began in Germany from 1920 [51]. The domestication and breeding history of rapeseed has a short period of time [52]. The genomic introgression of other Brassica species have resulted in abundant genetic variation within Brassica subgenomes, with substantial advantageous variations present across elite rapeseed breeding lines [53]. The rapeseed differentiation into three subtypes across distinct cultivation regions has enhanced diversity of the genetic pool [54]. Genotypic data effectively separated these three types into distinct populations, with significant phenotypic differences. The rich variations in rapeseed germplasms provide a biological basis for elite genes underlying important traits and for genetic improvement.

FT, OC, and yield-related traits are prioritized by breeders due to agronomic significance. These traits exhibit varying heritability levels (high to moderate), which provide insights into the genetic architecture of multigenic inheritance. FT is one of the most important breeding goals, which not only distinguished the subtypes of rapeseed, but also determine the final yield and quality [55]. FT is significantly correlated with silique development and bud differentiation [55, 56], which plays a key role with OC, SN, SL, and NSS. During breeding program, elite lines with suitable FT is crucial for yield and quality improvement [57]. Comprehensive analysis of the genetic architecture of FT, quality, and yield component traits using GWAS approach and the development of GP is of importance for breeding selection.

Benefiting from the development of biotechnology, high-throughput genomic and phenomic analysis can identify a large number of significant loci for complex traits. In our study, GWAS analysis of BLUP data for complex traits identified significant QTLs and underlying candidate genes. In this study, the FT trait had the highest number of QTLs, with *qFT.A02*, *qFT.A09*, and *qFT.C09* overlapping with the results of Wu *et al.* [57]. Notably, *qFT.A02*, a candidate gene (*BnaA02G0352400ZS*) encoding CDF1, was identified that represses *CO* expression [58] (Supplementary Table S5). Additionally, three tandem *FLC* genes (*BnaC09G0556100ZS, BnaC09G0556700ZS, BnaC09G0557000ZS*) were identified underlying *qFT.C09*. Among them, *BnaC09G0556100ZS* contributed significantly to *qFT.C09* by haplotype analysis (Supplementary Fig. S2). FLC is a key regulator of flowering and vernalization pathways. As a MADS-box transcription factor, it delays flowering by repressing genes like *FT* [59]. Prolonged cold exposure reduces *FLC* expression, releasing its repression on *FT* and *SOC1*, thus promoting flowering [60]. *FLC* gene polymorphisms also affect plant development [61]. The population differentiation coefficient analysis revealed that both flowering-related genes were located in highly differentiated regions shared by all three subgroups, suggesting that these candidate genes might play a key role in controlling FT in rapeseed. Except for FT, TSW showed a significantly positive correlation with SL, which also was found by Dong *et al.* [62]. *qSL.A09* for SL had an effect value of 1.96E-10 and was overlapped with *qTSW.A09* for TSW; both were located in regions of high differentiation (*Fst*) on chromosome A09. This region aligns with the interval identified by Shen *et al*. [63]. The QTL for SN, *qSN.C09*, is overlapped with *qFT.C09* for FT, with both being in a highly differentiated region on C09. Besides the big-effect significant QTLs (−log10(*P*) > 6) and variants, the relatively minor-effect QTLs and variants (−log10(*P*) > 3) were included in our study. Among the QTL, FT shared the most overlapping variants with other traits. A total of 1224 SNPs were detected to be simultaneously associating with two traits. Among those SNPs, 661 SNPs were associated with FT and another trait. There were 18 SNPs detected to be simultaneously associating with three traits, with 14 SNPs associated with FT and other two traits. These SNPs associated with two or three traits may exhibit pleiotropy, which should be given particular attention.

Integration of GP into breeding programs could accelerate and shorten breeding cycles and allow for multiple-trait improvement in rapeseed (Supplementary Fig. S3). As FT, quality and yield are all complex traits [64], it is a big challenge to GP and GS using seldom QTL. For genomic prediction, stronger relationships between input genotypic data and predicted traits lead to better accuracy. Filtering genotypic variants based on trait-specific effects can help build more accurate prediction models [33, 65]. High-throughput resequencing method was adopted to obtain >10 million genomic variants. However, the number of input features is a big challenge for effective model construction. To address this, three dimensionality reduction methods were applied to generate input features, and the prediction performances were compared with different models. PCA, CDS-originated variants, and GWAS-associated variants were used to derive three input feature sets. In this study, GWAS-associated variants produced more accurate phenotype prediction compared to random selection variants from PCA feature and CDS region variations. Using 10-fold cross-validation, prediction accuracy for six traits ranged from 83.7% to 95.6% with GWAS variations, from 22.96% to 77.82% with PCA features, and 26.67% to 80.72% with CDS variants. Compared with other traits, FT can be predicted with relatively higher accuracy. FT in rapeseed is a relatively stable and critical trait in different ecological regions. Breeders prioritize its selection during genetic improvement programs due to its high heritability and predictability. Genomic regions governing FT may exhibit conserved genetic architectures with distinct allelic differentiation, enabling robust GP across diverse genetic backgrounds. GP with GWAS association variants has proven to be highly effective in plants [66]. Compared to existing genomic selection studies [46–48, 67], increasing marker density allows for more accurate prediction by identifying effect variants with greater improvement. However, GP using genotype data with transcriptome and proteomics data can further improve the prediction accurancy [68]. When adopting GWAS results for genomic prediction, the optimizing prediction accuracy could be achieved with minor and major effect SNPs above relative low threshold and relatively low costs.

The best performing models were GBLUP and Bayes–Lasso, especially when using GWAS-associated variants as input features. Among ML models, GBDT was consistently stable across all traits. When PCA-reduced features and CDS variants were used as input, GBDT often performed best. Previous studies have extensively discussed the predictive efficacy of related models. However, differences among model accuracy are often negligible. Moreover, the predictive power is largely dependent on the genetic architecture of the traits [66, 68, 69]. For the models, the number of input feature is closely tied to the cost of genomic selection, favoring models that use as few markers as possible while maintaining high accuracy. In genome-wide selection models, prediction accuracy declines as the GWAS filtering threshold increases. Obviously, when the threshold is decreased from 5 to 3.5, more weakly associated variants are identified, and the prediction accuracy is significantly increased with 5.6%–36%. Especially for OC, SN, NSS, and TSW, the increases are 20%, 30%, 22%, and 36%, respectively. Therefore, adopting this method including minor effect variants into GP has a positive effect on improving prediction accuracy. When using 60 K chip sequencing data as the input genotype, a –log10(*P*) threshold of ~1.0 is required to reach saturation; however, the prediction accuracy remains relatively low [70]. When variants with effect value >3.5 were used, prediction accuracy for traits such as OC, SL, SN, NSS, and TSW was saturated. Increasing the number of variants could not significantly improve the prediction accuracy. When predicting complex traits, using relatively low-effect variants as supplementary markers is necessary for improving prediction accuracy. Thus, minor-effect variants are also important for GP.

The model selection in GP for complex traits plays a critical role in animal and plant breeding. The choice between traditional models and ML models depends on various factors, including the specific breeding context, dataset size, and the complexity of genetic architectures [71]. In some cases, such as wheat breeding, traditional models like GBLUP have shown superior predictive performance compared to ML models [72]. This is likely due to their robustness and efficiency in handling structured genetic data. In other cases, such as predicting bull conception rates, ML models like GBDT have achieved marginally higher accuracy than traditional models, though the difference may not always be statistically significant [73]. ML models generally require larger training datasets to effectively capture nonlinear genetic patterns, which are often present in complex traits. Traditional models (e.g. GBLUP, Bayes–Lasso) can perform well with smaller datasets and are computationally more efficient [74]. This is due to model self-regularization mechanisms and explicit modeling of genetic architecture, which effectively balance the bias–variance trade-off under limited data. In scenarios where the number of markers (p) far exceeds the sample size (*n*), traditional models tend to outperform ML models. In contrast, ML models (e.g. GBDT) require larger sample sizes to leverage expressive power without overfitting. ML models typically exhibit superior performance when sample sizes exceed 1000 individuals [75], as their capacity to capture complex interactions requires substantial training data. In this study, under the features of PCA and CDS, the prediction results of ML models are superior to those of traditional models. When the number of selected features is >5P, ML models achieve prediction accuracy comparable to traditional models. However, when increasing the number of GWAS markers (>4.5P, >4P, >3.5P, and >3P), traditional models show significant advantages over ML models. The comparative studies underscore that there is no one-size-fits-all model for genomic prediction. The optimal model depends on the specific breeding context, dataset characteristics, and the genetic complexity of the trait. As ML methods continue to evolve, their integration with traditional models could further enhance the accuracy and efficiency of genomic selection in both animal and plant breeding.

Throughout the history of rapeseed breeding, improving FT, OC, and yield has consistently ranked among the top breeding priorities. The regulation of these complex traits involves multigenes. This genetic complexity poses significant challenges to efforts aimed at enhancing rapeseed traits through breeding programs. However, genomic prediction allows for the rapid screening of rapeseed lines and identification of the most promising candidates that align with predefined breeding objectives. In this study, the GP models were constructed based on the genotypic and phenotypic data of FT, OC, and yield components. By optimizing genotypic features and models, the prediction accuracy was ultimately optimized for all the traits. Through the combination of prediction models for the FT, quality and yield, precise genome selection in rapeseed breeding was achieved. However, this study has certain limitations. The predictive capacity may be constrained by potential biases arising from the restricted scope and geographic origins of the germplasm resources, coupled with the absence of environmental interaction analyses for diverse genotypes. Nevertheless, the used genome variants and optimal prediction models demonstrate practical utility. Compared to whole-genome sequencing, custom SNP arrays significantly reduce expenses in genome prediction/selection strategies. Through integration with liquid-phase SNP array platforms and accelerated breeding technologies, these pipelines enable efficient pyramiding of superior alleles from global elite breeding resources, thereby significantly enhancing the genetic gain in rapeseed improvement programs.

## Materials and methods

### Plant materials and phenotypic data analysis

A total of 404 rapeseed lines were collected from different countries and regions, including Europe, Australia, Canada, and China [8, 55] (Supplementary Table S1). Field trials were carried out at the Yangluo Experimental Station of the Oil Crops Research Institute, Chinese Academy of Agricultural Sciences (30°N, 114°E) in two growth cycles of 2020–21 and 2022–23. The meteorological character for the two growth cycles was presented in Supplementary Table S4. All field trials were conducted under uniform management and cultivation conditions. The lines were planted in two randomized replicates, with three rows per plot and 18 plants per row, making a total of 54 plants per plot. Row spacing was 33 cm, and plant spacing within rows was 11 cm, with a planting density of 270 000 plants per hectare. All plants were cultivated in October, with harvesting in May of the next year. FT was recorded when 50% of the plants showed visible open flowers in the main inflorescence. OC in seeds was measured using a Foss 2500 near-infrared analyzer (Foss, Denmark). SN was counted randomly from 10 plants, and SL, NSS, and TSW were imaging-measured by Wanshen SC-G automatic system (http://www.wseen.com/ProductDetail.aspx?id=16&classid=28).

### Genotyping and evolutionary analysis

The original genome resequencing data of 124 rapeseed lines are stored on the GSA website (https://ngdc.cncb.ac.cn/gsa/), project ID CRA013310. An additional 280 lines were available on NCBI (https://www.ncbi.nlm.nih.gov/), project ID PRJNA476657. The sequencing depth was 10×, with an average sequencing amount of 12 GB, carried out on the Illumina HiSeq platform. Sentieon software was used to align reads to the rapeseed reference genome ZS11.v0 (ZS11 v20200127), and the HaplotypeCaller tool was used to detect gVCF files for each sample, followed by gVCF file merging using Sentieon. The GATK software was used to filter variant files with parameters set to QD < 2.0, FS > 50, MQ < 20, MQ Rank Sum > −12.5, and Read Pos Rank Sum > −8.0. SNP and InDel information were extracted and saved in VCF format. Quality control was performed using Plink software [76], with filtering criteria set to minor allele frequency >0.05 and missing data <10%, which resulted in high-quality SNPs and InDels for further analysis. Population structure analysis of the genotypic data was carried out using the CMplot package in R 3.3.3 to determine the number of variants in each 1-MB region of the genome and to plot variant density maps for assessing genome-wide variant coverage. Population structure analysis involved setting *R^2^* > 0.2 for LD pruning using Plink software. VCF2Dis software was used to calculate pairwise genetic distances based on VCF files, and the resulting distance matrix was uploaded to the FastMe2.0 website to create a phylogenetic tree, which was visualized using the iTOL website (https://itol.embl.de/). PCA of the genotypic data was performed in Plink, and the first two principal components were plotted. LD decay distances on chromosomes for three subgroups were estimated using PopLDdecay software [77]. *Fst* values were calculated using VCFtools, with a window size of 20 000 bp and a step size of 5000 bp.

### Multitrait association analysis

The BLUP algorithm in the lme4 package in R was utilized to calculate breeding values for the traits, which were subsequently used as input phenotypic values. EMMAX software was used to conduct a mixed linear model (MLM) analysis to assess the association between variants and corresponding traits, generating a kinship matrix to control for relatedness. The population structure (Q) and kinship matrix (K) were included in the model to reduce false positives due to relatedness. Manhattan and QQ plots were generated using the CMplot package to display GWAS results. The Manhattan plot shows the distribution of variants across the genome, where significant loci were identified by setting the significance threshold to –log10(*P*) > 6, while the QQ plot was used to evaluate the fit of observed *P*-values to the expected distribution. Map of SNPs and InDels distribution was also created using the CMplot package. Significant QTLs were identified using the Bonferroni-corrected threshold. LD-pruned genome files were filtered using Plink software, with a window size of 500 kb and a step size of 50 kb to filter out variants with LD decay *R^2^* > 0.2. The GEC software was used to calculate the number of effective measure (*M_e_*), and the significance threshold was set as one-tenth of *M_e_*. The effect intervals of GWAS were determined based on LD decay distances.

### Genotypic data dimensionality reduction

PCA of genotypic data was conducted using Plink software, and the cumulative variance explained by each principal component was calculated. Principal components explaining >95% of the variance were extracted as input features, and the first three principal components were plotted in a PCA clustering diagram. The GATK4 SelectVariants tool was used to extract all variants in the CDS regions from the population variant files. VCFtools was used to remove InDels information, leaving SNPs in the CDS regions. Random sampling of one variant every 20 variants was performed in R 3.3.3 to reduce dimensionality. Plink software was used to convert VCF files to 012 genotype format. In R 3.3.3, SNPs with effect values –log10(*P*) above thresholds of 3, 3.5, 4, 4.5, and 5 were extracted. Plink was used to extract corresponding variant information from VCF files, and VCFTOOLS was used to remove InDels information. Missing variants were imputed using BEAGLE software [78], and VCF files were converted to 0 1 2 genotype format using Plink.

### Prediction model construction

GBLUP and Bayes–Lasso models were imported using the BGLR package in R, with 10-fold cross-validation used to calculate prediction accuracy. In a Python 3.9 environment, ML models including Kernel Ridge Regression (KRR), Random Forest (RF), and GBDT were imported using the sklearn 1.0.2 library. eXtreme Gradient Boosting (XGBoost) and LightGBM models were imported using the xgboost 1.7.3 and lightgbm 4.1.0 packages, respectively. The scipy 1.11.1 library was used to calculate Pearson correlation coefficients between predicted values and GEBV to assess prediction accuracy, and 10-fold cross-validation was used to evaluate model accuracy. Bayesian hyperparameter optimization was performed using the Bayesian optimization 1.4.3 package. Regularization parameters for linear functions were set between 1 and 500, while the bandwidth range for the radial basis function was set between 0.00001 and 0.001. For tree models, the number of decision trees was set between 20 and 200, the maximum depth of weak learners was set between 5 and 25, and learning rate was set between 0.01 and 0.1. The initial number of observations was set to 20, followed by 280 iterations of optimization to select the optimal combination of hyperparameters.

## Supplementary Material

Web_Material_uhaf115

## Data Availability

The raw data of genome sequence could be publicly downloaded on the GSA website (https://ngdc.cncb.ac.cn/gsa/), project ID CRA013310, and on NCBI (https://www.ncbi.nlm.nih.gov/) ID PRJNA476657.
