# Supplementary material for: Optimization and application of genome prediction model in rapeseed: flowering time, yield components, and oil content as examples
Source: Hortic Res. 2025 Apr 30;12(8):uhaf115. doi: 10.1093/hr/uhaf115 (PMC12258037; doi:10.1093/hr/uhaf115)
Supplement: Web_Material_uhaf115 [file web_material_uhaf115.zip › 02 Supplementary Figure 1-3.docx]

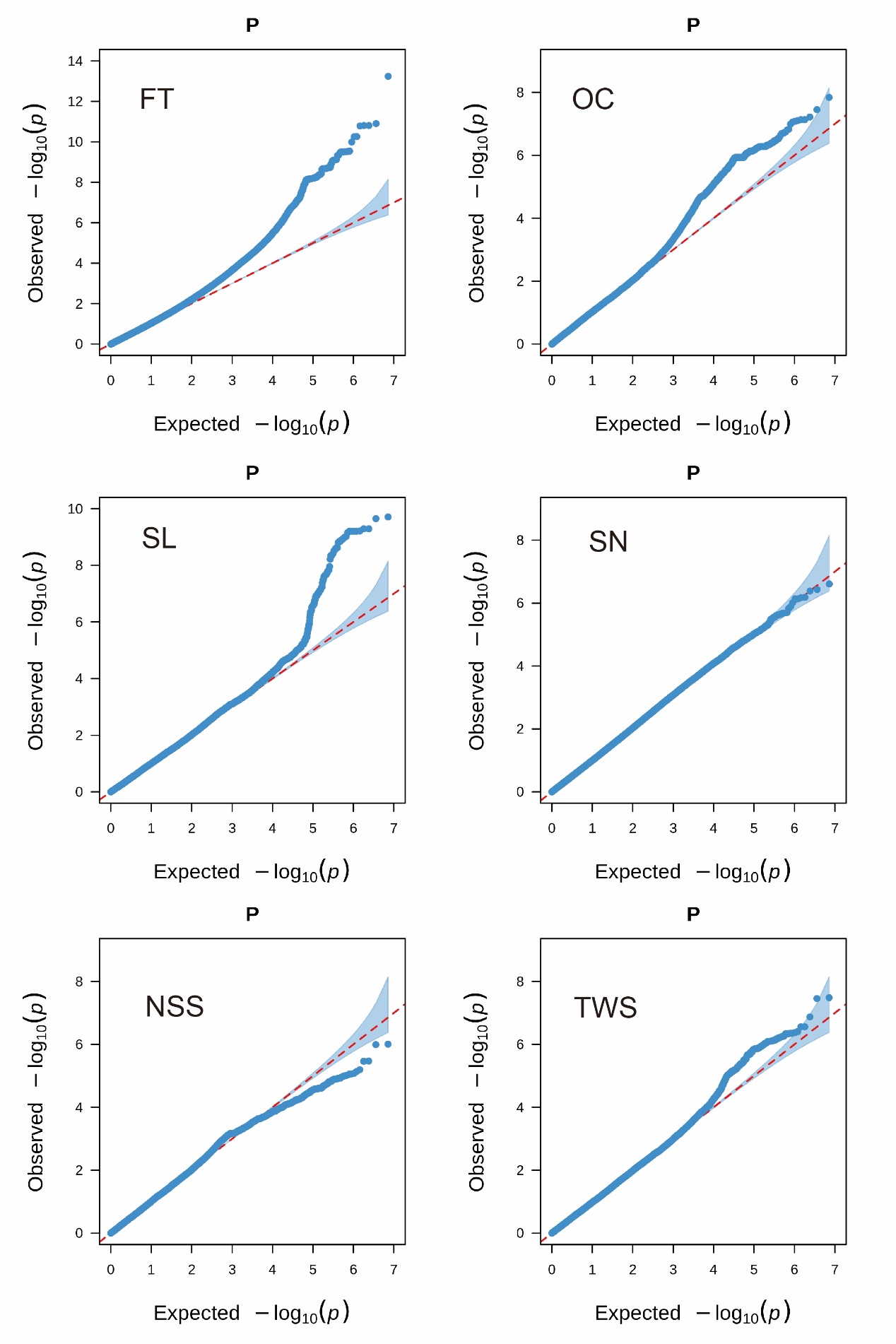


Supplementary figure 1. QQ plots from GWAS analysis for six traits.


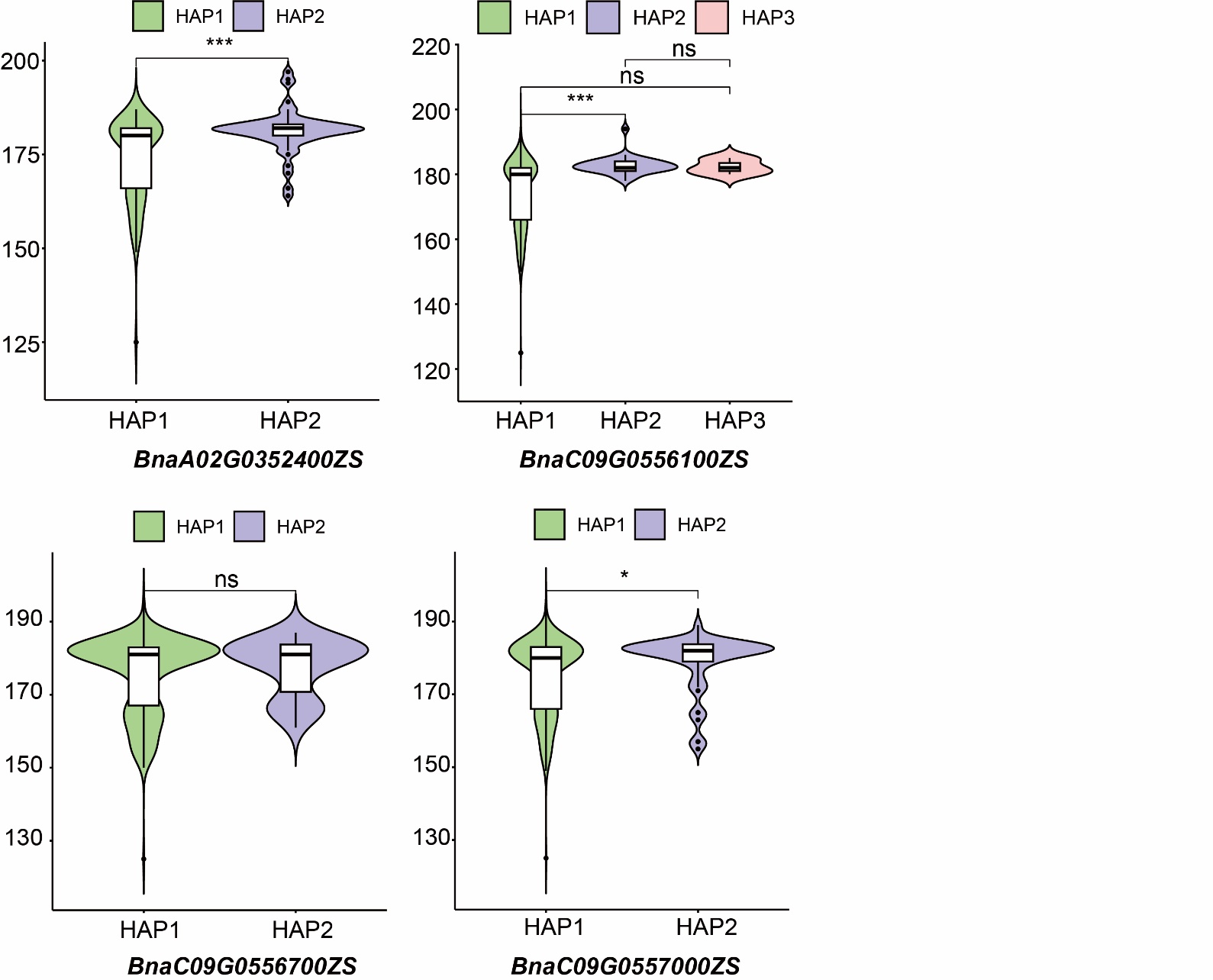


Supplementary figure 2. Haplotype analyse for the four candidate genes including *CDF1* (*BnaA02G0352400ZS*), *FLC* (*BnaC09G0556100ZS, BnaC09G0556700ZS, BnaC09G0557000ZS*) underlying FT QTL. Statistical significance of haplotype effects is determined by a T-test, with *, *p* < 0.05; **, *p* < 0.01; and ***, *p* < 0.001.


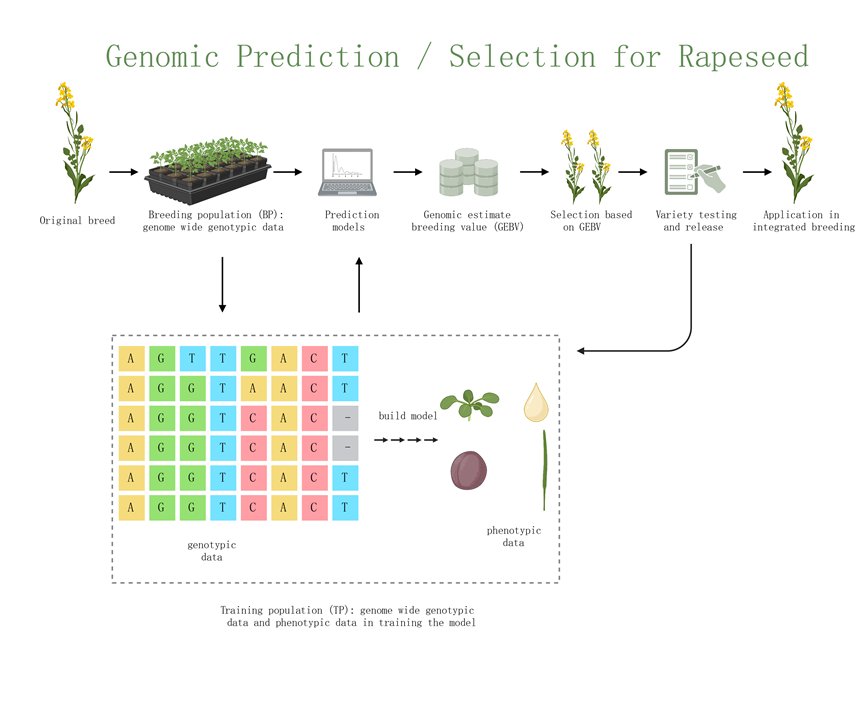


Supplementary figure 3. Genomic prediction/selection for rapeseed breeding.
